# Testosterone and gonadotropins but not SHBG vary with CKD stages in young and middle aged men

**DOI:** 10.1186/s12610-015-0027-y

**Published:** 2015-12-02

**Authors:** Britta Hylander, Mikael Lehtihet

**Affiliations:** Department of Nephrology, Karolinska Institute and Karolinska University Hospital, Solna, Stockholm, S-17173 Sweden; Department of Endocrinology, Metabolism and Diabetes, C2:94, Karolinska Institutet at Karolinska University Hospital, Huddinge, Stockholm, S-141 86 Sweden

**Keywords:** Chronic kidney disease, Hypogonadism, Testosterone level, maladie rénale chronique/hypogonadisme/taux de testostérone

## Abstract

**Background:**

The aim of this study was to assess the effects chronic kidney disease (CKD) had on sex hormones and lipids in a subgroup of men between 18 and 50 years old with CKD 1–5 stage without diabetes and not treated with hemodialysis.

**Methods:**

Data were collected from 101 men with different CKD stages.

**Results:**

Higher CKD stage (lower function) had a significant negative linear trend on total testosterone level (*p* < 0.01) and free testosterone level (*p* < 0.01), with a significant increase of luteinizing hormone (LH) (*p* < 0.01), and prolactin (*p* < 0.01), while SHBG remained unchanged between the CKD stages. Triglycerides but not total cholesterol, HDL –cholesterol or LDL-cholesterol increased with higher CKD stage. A negative correlation was observed between BMI, SHBG and free testosterone (*p* < 0.01 for both) but not with other sex hormones. Age *per se* was related to a significant decrease of total and free testosterone level (*p* < 0.01 for both) even after correction for BMI.

Decreased levels of total testosterone and estimated free testosterone levels had a significant correlation with an increased level of triglyceride levels (*p* <0.01).

**Conclusions:**

Our results indicate that CKD stage *per se* is a factor affecting testosterone levels in combination with age in men between 18 and 50 years old with CKD 1–5 stage, not treated with hemodialysis.

With increased CKD stage there was a significant increase in LH level and a pattern of hypergonadotropic hypogonadism. SHBG remained unchanged between the CKD stages.

**Electronic supplementary material:**

The online version of this article (doi:10.1186/s12610-015-0027-y) contains supplementary material, which is available to authorized users.

## Résumé

**Objectifs:**

Le but de cette étude était d’évaluer les effets d’une maladie rénale chronique (MRC) sur les hormones sexuelles et les lipides dans une sous-population d’hommes âgés de 18 à 50 ans porteurs d’une MRC de stade 1-5, non diabétiques et non traités par hémodialyse.

**Méthodes:**

Les données ont été obtenues chez 101 hommes qui présentaient différents stades de MRC.

**Résultats:**

Un stade plus élevé de MRC (fonction plus réduite) a une tendance linéaire négative sur les taux de testostérone totale (p<0,01) et de testostérone libre (p<0,01), avec une augmentation significative de la LH (p<0,01) et de la prolactine (p<0,01), alors que les taux de SHBG ne diffèrent pas entre les stades. Les triglycérides augmentent avec les stades plus élevés de MRC, ce qui n’est pas le cas du cholestérol total, du cholestérol HDL, ou du cholestérol LDL. L’IMC est négativement corrélé à la SHBG (p<0,01) et à la testostérone libre (p<0,01), mais n’est pas corrélé aux autres hormones sexuelles. L’âge per se est lié à une diminution significative des taux de testostérone totale (p<0,01) et de testostérone libre (p<0,01), corrélation qui persiste après ajustement sur l’IMC. Des taux diminués de testostérone totale et de testostérone libre estimée sont significativement corrélés à un niveau augmenté des taux de triglycérides (p<0,01).

**Conclusions:**

Nos résultats indiquent que le stade de la MRC per se est un facteur qui affecte les taux de testostérone en combinaison avec l’âge chez les hommes de 18 à 50 ans porteurs d’une MRC de stade 1-5 et non traités par hémodialyse.

L’élévation du stade de MRC est associée à une augmentation significative du taux de LH et à un profil d’hypogonadisme hypergonadotrophique. La SHBG n’est pas modifiée par le stade de MRC.

## Background

Chronic kidney disease (CKD) is found in more than 10 % of the general population [1, 2]. It is well recognized that cardiovascular diseases (CVD) are linked to CKD [3], and that chronic kidney disease is recognized as an independent risk factor for premature CVD [3]. The exact role of androgens in the development of cardiovascular and CKD is still unclear.

In men with type 2 diabetes mellitus suboptimal testosterone concentration is a common finding in one-third of the men along with a normal LH [4]. And presence of type 2 diabetes and CKD increase the prevalence of hypogonadism from 5 % to 26 % [4]. Data from meta-analysis have also shown that men with non-diabetic CKD have a faster progression to end stage renal disease (ESRD) compared to female, although other studies did not identify gender as a risk factor for CKD or for CKD progression [5, 6]. In men with CKD gonadal dysfunction with elevation of serum gonadotropin concentration is a frequent finding, affecting 26–66 % of men with different stages of CKD [7].

Subfertility is also a common problem in men with CKD and we have previously shown that CKD stage *per se* is a factor determining the number of spermatozoa available in epididymis for ejaculation. This was in part independent of age-related decrease of testosterone level and BMI in a subgroup of men between 18 and 50 years old with CKD 1–5 stage, not treated with hemodialysis [8].

Hypergonadotropic hypogonadism is a well established hormonal derangement associated with CKD [9]. In a population free from renal disease, testosterone deficiency is suggested to take part in the atherosclerotic process. Emerging evidence indicates that androgens may provide a protective effect against the development and/or progression of atherosclerosis in men [10]. Hypogonadism in men with CKD not yet on dialysis, has been associated with arterial stiffness and endothelial dysfunction [11] supporting the theory that hypogonadism could be a key link with CVD in this group of men [12]. However it cannot be excluded that the atherosclerotic process significantly increases as a consequence of progression of the kidney disease (e.g. low testosterone levels in CKD may therefore be an adaptive mechanism during progression) rather than as a consequence of low testosterone level per se [13].

It is also possible that prescribed medications to patients with CKD could interfere directly with synthesis of sex hormones e.g. statins, angiotensin-converting enzyme (ACE) inhibitors and angiotensin II receptor blockers (ARBs) [7, 14, 15]. Several expert groups have presented guidelines for the diagnosis of testosterone deficiency [16, 17]. Those guidelines recommend that the diagnosis of hypogonadism should only be made in men with characteristic symptoms or signs of testosterone deficiency in combination with a documented low serum testosterone level.

The most widely accepted parameters to establish the presence of hypogonadism is the measurement of total serum testosterone. There are no generally accepted lower limits of normal total testosterone levels. There is, however, general agreement that patients with a total serum testosterone level above 12 nmol/L generally do not benefit from treatment [17].

Most studies of sex hormones in men with CKD have been focused on patients with end stage renal disease (CKD 5 without dialysis) and on patients on hemodialysis, and there is little information about sex hormone level development and serum lipid profiles throughout the different CKD stages. This study analyzes aspects of sex hormone levels and lipids, among a subgroup of Swedish men between 18–50 years old in CKD 1–5 stage, without diabetes mellitus and not treated with hemodialysis.

## Methods

### Subjects

Patients with different CKD stages aged 18–50 years from the Department of Nephrology, Karolinska University Hospital, Stockholm, Sweden were recruited from December 2012 to December 2013 and divided into five groups according to their stage of renal impairment (CKD 1-CKD 5). Staging of CKD was defined according to the presence or absence of kidney damage and level of kidney function, irrespective of type of kidney diagnosis [18], according to Table 1. The CKD stage was based on Creatinine–Cystatin C Equation) for Estimating Glomerular Filtration Rate (eGFR) [19].

**Table 1:** Stages of chronic kidney disease (CKD) was defined according to the presence or absence of kidney damage and level of kidney function, irrespective of the type of kidney disease (diagnos) [43]

Stage	Description	GFR (ml/min per 1.73 m^2^)
1	Kidney damage with normal or increased GFR	≥90
2	Kidney damage with mild decreased GFR	60 to 89
3	Moderate decreased GFR	30 to 59
4	Severe decreased GFR	15 to 29
5	Kidney failure	<15 or dialysis

GFR values are normalized to an average surface area of 1.73 m^2^

Of the 145 men invited 101 fulfilled inclusion criteria and volunteered to provide fasting blood samples. In Tables 2 and 3 characteristics of the participants are included. Participants were not included if they were smokers or former smokers (>3 months), had type 1 or type 2 diabetes mellitus, had a previous renal transplantation, or were treated with testosterone replacement therapy. Patients with diabetes mellitus were excluded because both forms of diabetes may be associated with hypercholesterolemia and hypertriglyceridemia at hyperglycemia [20].

**Table 2:** Baseline clinical characteristics of participants in stage CKD 1–5

	CKD 1	CKD 2	CKD 3	CKD 4	CKD 5	p
*N* = 101	23	20	27	13	18	
Age (year)	34.0 ± 9.1	40.1 ± 6.1	41,7 ± 6,4	42.1 ± 5.95	38.5 ± 8.32	n.s.
BMI (kg/m^2^)	26.1 ± 5.2	25.9 ± 5.3	25.1 ± 3.6	27.3 ± 5.33	25.2 ± 2.89	n.s.
Hemoglobin (g/L)	149.9 ± 7.2	146.4 ± 9.1	133.8 ± 13.9	121.4 ± 12.1	111.6 ± 13.3	<0.001
Creatinine ( μmol/L)	81.6 ± 11.2	110.7 ± 15.2	153.1 ± 38.9	387 ± 162	633.1 ± 203	<0.001
Cystatin C (mg/L)	4.26 ± 0.29	2.44 ± 0.13	1.66 ± 0.12	0.98 ± 0.05	0.84 ± 0.05	<0.001
eGFR	92.6 ± 1.5	77.1 ± 9.5	47.9 ± 10.0	21.1 ± 5.4	12.1 ± 2.0	<0.001

CKD staging was tested with one way ANOVA. Data were considered statistically significant at *P* < 0.05CKD stage was based on Creatinine–Cystatin C Equation) for Estimating GFR (eGFR) [19]

**Table 3:** Diagnosis of participants in stage CKD 1–5

Diagnosis	CKD 1	CKD 2	CKD 3	CKD 4	CKD 5
* N* = 101	23	20	27	13	18
Glomerular disease	11	13	15	7	11
Hypertensive nephrosclerosis	2	2	3	1	2
Tubulointerstial disease	2	2	3	1	1
Vascular disease	3	1	1	1	3
Polycystic kidney disease	5	2	5	3	1
Medications					
Statins	5	3	14	6	8
ACE inhibitors	11	10	13	8	9
Angiotensin receptor blockers	2	11	9	8	10
Diuretic	0	3	5	6	10
B-blockers	3	4	6	7	10
Calcium channel blockers	4	5	13	6	7

All participants gave written and oral informed consent to participate, and the study was approved by the Ethics Committee of Karolinska Institute.

### Assays

All samples were taken in morning (07.00-09.00 a.m.) after an overnight fast (12 hrs).

Total testosterone level was measured with a chemiluminescent immunoassay for quantitative determination of total testosterone level in human serum and plasma using the Access Imunoassay System (Beckman Coulter). The intra-assay and inter-assay coefficients of variation for testosterone were less than 5.0 %. Free serum testosterone was calculated by the method of Vermeulen [21].

LH and FSH were determined with an AutoDELFIA hLH assay (PerkinElmer Life and Analytical Sciences, Turku, Finland) two-site immunoradiometric assay. The intra-assay and inter-assay coefficients of variation were 1.9 and 2.2 % respectively for LH and 2.2 and 3.5 % for FSH. SHBG and prolactin were measured with a paramagnetic particle chemiluminescent immunoassay (Access SHBG assay UniCel DxI 800, Beckman Coulter, Inc, USA). The intra-assay and inter-assay coefficients of variation for SHBG were 4.0 and 5. 5 % respectively, and for prolactin 3.5 and 5.0 %. Plasma glucose concentration was measured by a glucose oxydase method (Glukos HK, Modular P, Roche Diagnostics, Indianapolis, IN). The intra-assay and inter-assay coefficients of variation for glucose were 1.2 and 1.9 % respectively.

Total cholesterol, High-density lipoprotein (HDL)-cholesterol, Low-density lipoprotein (LDL) – cholesterol, creatinine and cystatin C were measured by the routine chemistry accredited laboratory at the Karolinska University Hospital (Modular P, Roche Diagnostics, Mannheim). LDL-cholesterol was calculated according to the Friedewald formula [22]. All participants had plasma triglycerides < 4.5 mmol /L (the upper limit for estimates with the formula). Reference range for healthy men between 18–50 years of age is included in the Additional file 1: Table S1.

## Statistical analysis

All data shown are expressed as means ± SD. The normality was tested with a probability plot and the Kolmogorov-Smirnov one-sample test. Log transformed values were used for prolactin, SHBG and triglycerides levels in the analysis and then back transformed for data presentation. One way Analysis of Variance (ANOVA) was used to test the differences between CKD stages for age, BMI, Hemoglobin, creatinine, eGFR, sex hormones and lipids. A Spearman´s rank test was used to determine univariate correlations between variables. Multiple linear regression analyses were performed with BMI and age as covariates. Data were considered statistically significant at *p* < 0. 05.

Statistical analyses were performed using Statistica, Statsoft version 10.0 (Tulsa, OK, USA).

## Results

### Clinical characteristics

The clinical characteristics of the 101 men who were included and had accepted to participate in the study are shown in Tables 2 and 3. The group of men enrolled in this study did not differ significantly in age or BMI between the different five CKD stages.

### Sex hormone level in serum

There was a significant decrease in total testosterone level and estimated free testosterone with CKD stages, *p* <0.01 for trend for both. LH and prolactin levels increased significantly, *p* <0.01. FSH showed a tendency to increase, although not significantly. No significant differences were observed for SHBG levels among the different CKD stages, Table 4.

**Table 4:** Comparison of the levels of sex hormones and lipid levels in serum in participants in stage CKD 1–5

	CKD 1	CKD 2	CKD 3	CKD 4	CKD 5	p
*N* = 101	23	20	27	13	18	
S- Testosterone (nmol/L)	14.89 ± 3.91	14.10 ± 4.15	13.22 ± 3.81	10.81 ± 4.53	8.82 ± 3.81	<0.01
S-SHBG (nmol/L)	29.56 ± 12.73	35.50 ± 13.03	34.55 ± 13.51	33.07 ± 12.9	28.55 ± 13.47	ns
Free testosterone nmol/L	0.33 ± 0.10	0.28 ± 0.06	0.25 ± 0.05	0.19 ± 0.07	0.19 ± 0.08	<0.01
S-LH (U/L)	4.16 ± 2.07	3.33 ± 1.40	6.77 ± 4.22	6.50 ± 2.71	9.43 ± 7.60	<0.01
S-FSH (U/L)	3.67 ± 2.65	3.67 ± 2.06	6.85 ± 4.04	5.21 ± 4.82	13.51 ± 6.98	ns
S-Prolactin (μg/L)	7.76 ± 2.04	8.54 ± 3.36	9.24 ± 2.47	16.50 ± 12.54	17.41 ± 2.13	<0.01
S-Cholesterol (mmol/L)	5.04 ± 1.14	5.03 ± 1.65	4.85 ± 1.06	4.72 ± 0.72	5.20 ± 1.30	n.s.
S-HDL (mmol/L)	1.20 ± 0.31	1.25 ± 0.24	1.2 ± 0.29	1.09 ± 0.24	1.24 ± 0.48	n.s.
S-LDL (mmol/L)	2.98 ± 0.99	3.34 ± 1.08	3.02 ± 0.97	2.70 ± 0.71	3.03 ± 1.21	n.s.
S-Tg (mmol/L)	1.65 ± 1.12	1.17 ± 0.66	1.33 ± 0.68	2.17 ± 1.43	2.61 ± 1.52	<0.01
S-glucose (mmol/L)	5.13 ± 0.59	5.07 ± 0.45	5.18 ± 0.67	4.80 ± 0.67	5.18 ± 0.62	n.s.

Values reported are mean ± SD. Linear trend between sex hormones and CKD staging was tested with one way ANOVA. Data were considered statistically significant at *P* < 0.05. The conversion factor for prolactin from mass to units is 21.2. ((μg/L) x 21.2 = mIU/L)

### Serum lipid profile

In men with CKD stage 1–5 a significant increase in triglycerides levels was seen with increased CKD stage, *p* < 0.01. No significant differences were observed for total cholesterol, HDL-cholesterol or LDL-cholesterol levels among the different CKD stages, Table 4.

### Relationships between testosterone and lipids

The effect of decreased total testosterone levels on lipid parameters had a significant correlation with level of triglycerides (r = − 0. 42, *p* <0.001) and HDL –cholesterol (r =0.25, *p* = 0.01), Table 5. A decrease although not significant was also seen for total cholesterol level but not for LDL-cholesterol, Table 5. Free testosterone correlated with level of triglycerides but not with HDL-cholesterol. A borderline significant decrease was seen for total cholesterol, Table 5.

**Table 5:** Multiple linear regression analyses were performed with BMI and age as covariates

	Total testosterone		Free testosterone	
	Spearman R	*P*-value	Spearman R	*P*-value
Cholesterol	−0.19	0.053	−0.19	0.05
HDL-cholesterol	0.25	0.01	0.10	0.27
LDL-cholesterol	−0.06	0.55	−0.16	0.09
Triglycerides	−0.42	0.001	−0.23	0.02

Data were considered statistically significant at *P* < 0. 05

### Effect of BMI and age on sex hormones

In this study BMI had no significant correlation with total testosterone, LH, FSH or prolactin. A significant correlation with free testosterone was observed, Table 5. There was a negative significant correlation with SHBG (*p* < 0.01), Table 5. This significant correlation persisted after correction for age. Age was correlated to a decrease in total testosterone level and free testosterone level (*p* < 0.01) even after correction for BMI.

## Discussion

The association of different stages of CKD with sex hormones has been examined in several previous studies [11, 23]. As far as we know most studies in men with CKD have been performed in older patients or patients with end stage renal disease. It has been shown previously that there is a negative correlation between endogenous testosterone and CKD stage 1–5 [7, 11]. The mechanism for this is likely to involve, at least in some part some alteration or derangement of the male reproductive hormone profile [9]. This supports our findings of a significant decrease in testosterone level with increased CKD stages even in younger men and middle-aged men.

Whether decreased testosterone level is a result of degradation of uremic metabolites accumulated mainly in testes and affecting the Leydig cells as a result of progressive kidney disease or an inhibition of cAMP production associated with inhibition of 125I-human chorionic gonadotropin binding of the luteinizing hormone receptor in Leydig cells [23] is still unknown. A previous study has supported the theory that chronic kidney disease mainly affect testes by a defect in 17 ~ −hydroxysteroid dehydrogenase evidenced by a decrease in testosterone/androstenedione ratio at progressive CKD and a lack of correction by hCG administration [24]. With higher CKD stages there was a significant increase in the LH level and the development of a pattern of hypergonadotropic hypogonadism, which indicates that uremic metabolites secondary to the increased CKD stage affect testes more than the hypothalamic or pituitary function. Alternatively the degradation of uremic metabolites in the hypothalamic or pituitary region is faster and more pronounced than in testes. Previous studies have shown that hemodialysis does not improve the function of the HPT axis [25] but that renal transplantation may reverse the uremic damage to testicular function, suggesting that clearance of uremic metabolites is insufficient on hemodialysis treatment.

A novel finding was that SHBG level was unchanged between CKD stages, supporting the theory that also bioavailable testosterone decreases with CKD staging. The unchanged SHBG level does not support previous finding that estradiol level increases in men with CKD [26]. A high concomitant estradiol level is a common finding in men with CKD and gives a concomitant increase of SHBG level [26, 27]. However we cannot exclude that estradiol levels increase with CKD stages and then compensate the effect of decreasing testosterone on SHBG levels. The discrepancy between our results and previous studies could in part be explained by exclusion of patients with diabetes mellitus included in this study, and also that there were no patients with high BMI included, thus the metabolic impact could be minor in our study.

Of additional interest is insulin even among CKD patients without manifest or overt diabetes mellitus. Clearance of insulin is reduced with progressive CKD stage [28] and insulin resistance appears at an earlier stage of CKD [28] . Induction of insulin resistance in podocytes leads to glomerulosclerosis in animal models and development of CKD in observational human studies [29]. Elevated insulin level also produces a decrease in the hepatic production of SHBG [30] which in part can explain our results.

SHBG is also down regulated by proinflammatory cytokines that are associated with the prevalence and severity of CKD [31]. And SHBG levels can therefore represent a sensitive indicator of low-grade inflammation.

Hyperprolactinemia has been shown to affect at least 30 % of patients with CKD [32] and is the consequence of both reduced renal clearance [33] and increased production [34]. Prolactin might influence the metabolism of SHBG [35] by its inhibiting hormonal influences and decrease of SHBG in hyperprolactinemia [35]. In our study and a previous study of men without renal diseases [36] there was no correlation between SHBG and prolactin, but that does not exclude a correlation at a higher threshold level.

Hyperprolactinemia in CKD may be a contributing factor in the atherosclerotic process [37]. Increased expression of prolactin receptors has been found in human atherosclerotic plaques [38] and may be contributing factor to vascular derangements per se [37]. The lack of significant changes in SHBG levels among the participants in our study could be multifactorial due to medications, lifestyle comorbidity and dietary factors [16, 39]. With nephrotic syndrome as a condition associated with alternations in SHBG concentration [16].

In the present study there was a significant negative correlation with triglyceride levels and CKD stages. Furthermore decreased testosterone levels were seen in more severe CKD stages with more than 58 percent increase of triglycerides in CKD stage 5 compared with CKD 1 stage. It has previously been shown that a higher triglyceride level contributes to a more rapid decline in renal function in a non-diabetic patient cohort with CKD [40].

In a previous study by Gungor et al. [41] a negative correlation was seen between total cholesterol, triglyceride and testosterone levels in the cases on the chronic hemodialysis schedule. It is likely that we could not find a significant correlation between CKD staging and total cholesterol due to the small number of participants and high number of patients using antihyperlipidemic agents.

Our study has several limitations. Firstly, this was a cross-section observational study performed in a small cohort of patients with different diagnoses and CKD stages, and different medical treatment including statins, Table 2. Although the most prominent effects attributable to statin therapy are the potent LDL-cholesterol lowering properties, it is also well established that statins significantly reduce triglycerides [42]. It can therefore not be excluded that some of the difference observed could be related to statin treatment. However statin treatment among patients was more common with increased CKD staging, Table 2.

There was a negative correlation between age and decreased testosterone level and also between triglycerides and the testosterone level. No correlation was seen with BMI and sex hormones, except a negative correlation with SHBG. This might be explained by the limited number of participants and lack of overweight and obese participants included.

## Conclusions

CKD stage *per se* is a factor determining testosterone level together with age. A decreased testosterone level is also correlated with an increased level of triglycerides but not with cholesterol SHBG remained unchanged between the CKD stages.. With an increased CKD stage there was a significant increase in LH level, which indicates that uremic metabolites secondary to increased CKD stage in men between 18 and 50 years old with CKD 1–5 stage, not treated with hemodialysis affect testes more than the hypothalamic or pituitary function.

## Additional file

Additional file 1: Table S1. A summary of the reference range for healthy men between 18–50 years of age. The conversion factor for prolactin from mass to units is 21.2. ((μg/L) x 21.2 = mIU/L). (DOC 33 kb)

## Abbreviations

CKD: Chronic kidney disease
CVD: Cardiovascular disease
ESRD: End stage renal disease
ANOVA: Analysis of variance
LH: luteinizing hormone
SHBG: Sex hormone-binding globulin
FSH: Follicle stimulating hormone
S-T: S-Testosterone
eGFR: estimated Glomerular Filtration Rate
ACE inhibitors: Angiotensin-converting enzyme inhibitor
ARB: Angiotensin II receptor blockers
