# Supplementary material for: Testosterone and gonadotropins but not SHBG vary with CKD stages in young and middle aged men
Source: Basic Clin Androl. 2015 Dec 2;25:9. doi: 10.1186/s12610-015-0027-y (PMC4668662; doi:10.1186/s12610-015-0027-y)
Supplement: Additional file 1: Table S1. — A summary of the reference range for healthy men between 18–50 years of age. The conversion factor for prolactin from mass to units is 21.2. ((μg/L) x 21.2 = mIU/L). (DOC 33 kb) [file 12610_2015_27_MOESM1_ESM.doc]

Additional file 1: Table S1

| Analyses | Reference  Range |
| --- | --- |
| S- Testosterone (nmol/L) | 10-30 |
| S-SHBG (nmol/L) | 15-56 |
| Free testosterone  nmol/L | 0.17±0.72 |
| S-LH (U/L) | 1.2–9.6 |
| S-FSH (U/L) | 1.0–12.5 |
| S-Prolactin (µg/L) | 2.6–13 |
| S-Cholesterol  (mmol/L) | 2.9–6.1 |
| S-HDL (mmol/L) | 0.8-2.1 |
| S-LDL (mmol/L) | 1.2–4.3 |
| S-Tg (mmol/L) | 0.45-2.6 |
| S-glucose (mmol/L) | 4-6 |
| Hemoglobin (g/L) | 134-170 |
| Creatinine ( µmol/L) | < 100 |
| Cystacin C (mg/L) | < 0.99 |
